# Unidentified Intracranial Foreign Body in an Epileptic Child: Infanticide or Child Abuse?

**DOI:** 10.7759/cureus.47167

**Published:** 2023-10-17

**Authors:** Zeeshan Ullah, Muneeb ur Rehman, Amna Akbar, Sabhat Tasneem, Sarosh Khan Jadoon

**Affiliations:** 1 Neurology, Lady Reading Hospital, Peshawar, PAK; 2 Emergency and Accident, District Headquarter Jehlum, Muzaffarabad, PAK; 3 Public Health Sciences, Health Services Academy, Islamabad, PAK; 4 General Surgery, Combined Military Hospital, Muzaffarabad, PAK

**Keywords:** penetrating injuries, child abuse, infanticide, recurrent seizures, intracranial foreign body

## Abstract

Although intracranial foreign bodies are typically associated with penetrating injuries or surgical interventions, they can also occur as a result of rare instances of child abuse. Enduring such abuse and neglect as an infant can lead to life-long neurological problems, developmental delays, and impairments. The present case involved a 14-year-old male adolescent who was brought to the emergency room due to recurrent generalized tonic seizures. A computed tomography (CT) scan revealed a ring-like metallic object within the right temporal lobe. The neurosurgeon declined the surgical removal of the object due to its position and orientation, as well as the patient’s guardian’s refusal to consent to surgery. Instead, drug treatment and care are advised. In infants, foreign objects are typically inserted through cranial sutures, fontanelles and less frequently into the orbits, often with the intention of harming unwanted children. However, no history of such an attempt is present in this case. The incidental discovery of intracranial foreign bodies typically occurs during investigations when patients present with neurological symptoms such as epileptic seizures (foreign body-induced epilepsy). The selection of an ideal treatment regimen is often challenging in such cases. If a patient can be effectively treated with drugs, surgical removal is usually avoided.

## Introduction

Non-missile intracerebral penetrative injuries resulting from intracranial foreign bodies are rare in neurosurgery. Most traumatic wounds are the result of malicious crimes, self-inflicted injuries, or workplace negligence. The discovery of foreign objects in the cranial cavity, including blades, pencils, nails, and wood fragments, is a rare phenomenon [1]. Generally, intracranial foreign bodies are caused by trauma that penetrates cranial bones, orbits, and ears. Surgical instruments are rarely used in brain surgeries. Intracranial needles have also been documented [2]. Intracerebral objects of unknown origin may have been introduced into childhood before the closure of fontanelles [3]. This type of occurrence may have been caused by another child piercing a baby with metallic objects or by an adult intentionally harming an unwanted child.

These foreign bodies are often discovered during evaluation when patients present with head injuries or neurological symptoms such as headaches, seizures, and intracranial infections [4]. The management of intracranial metallic foreign bodies found incidentally can be controversial, and some neurosurgeons may choose nonsurgical treatments if the patient is asymptomatic [1]. This report highlights a unique case of an incidentally discovered intracranial metallic ring-like object in a male adolescent who had experienced seizures in the past. This is the first case report of a ring-like metallic object within a cranium, as per our knowledge.

## Case presentation

An adolescent male of 14 years was brought to the emergency department at our hospital in Peshawar, Pakistan, from a rural area near Peshawar. He presented with a history of recurrent episodes of convulsions over the course of the past two days. He had three consecutive episodes of generalized tonic-colonic seizures (GTCS), accompanied by frothing at the mouth and loss of consciousness. Each episode lasted between three and six minutes, with no signs of urinary or fecal incontinence. The patient had no history of fever, headache, vomiting, trauma, skull fracture, or craniotomy or gunshot surgery. Medical history revealed that he experienced his first episode of convulsions at the age of five months and was currently taking oral valproic acid (250 mg DB dose) for seizure management. Despite initially responding well to medication, the patient was recently non-compliant with treatment, leading to uncontrolled seizures. His birth history was unremarkable, and no significant delays in developmental milestones were reported. Family history was also negative for any neurological disorder.

**Figure 1:**
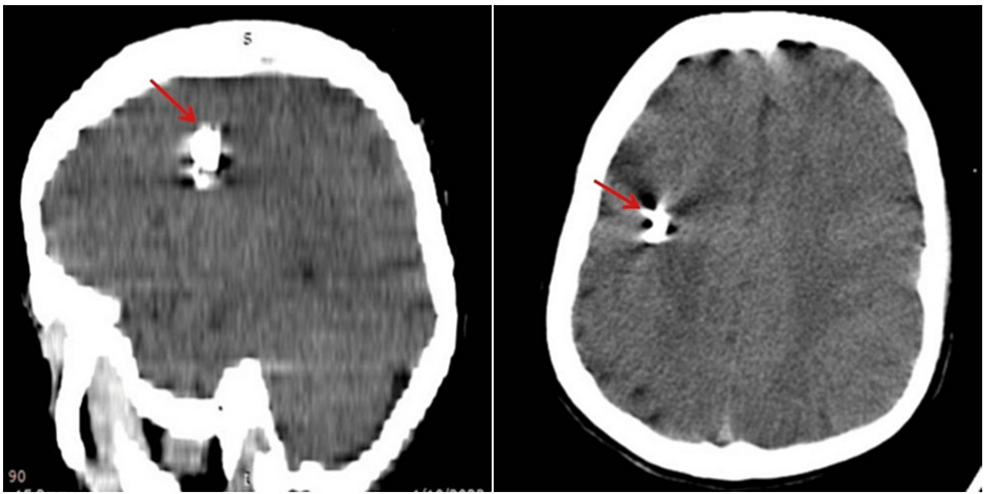
CT brain CT brain sagittal and axial views show foreign objects adjacent to the anterior fontanelle in the right temporal lobe.

**Figure 2:**
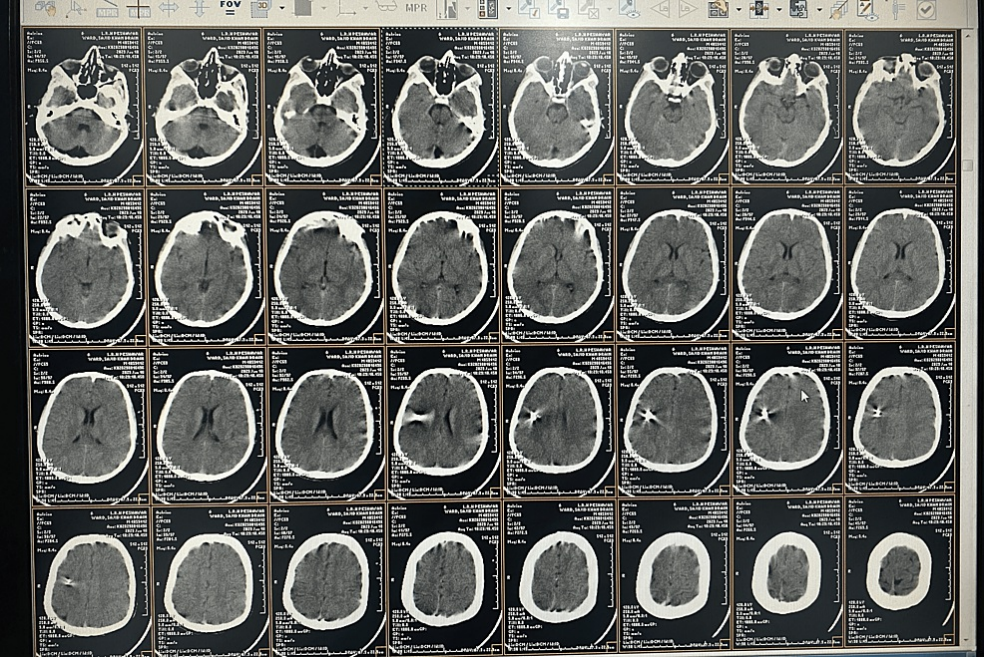
Computed tomography image The foreign body with a ring shape and metallic glare can be seen on the CT scan in the frontal lobe region near the cortex.

A diagnosis of status epilepticus was established, and treatment was initiated with an intravenous injection of diazepam (10 mg). The patient did not respond to diazepam and experienced another GTCS episode. A loading dose of valproic acid (1.5 g) was then administered as an intravenous infusion, leading to a positive response, and the patient regained consciousness. After his condition stabilized, he was admitted to the neurology unit for advanced assessment. The patient was oriented in time and consciousness, as per the neurological assessment. Neurological, motor, and sensory examination findings were unremarkable, and there were no signs of physical injury, including wounds, scars, abrasions, or fractures of the skull. The patient had no evidence of cognitive impairment. The baseline investigations yielded results that were within the normal limits. Computed tomography (CT) revealed a hyper-dense lesion with metallic glare in the right frontal lobe (Figure 2), suggesting the presence of a metallic foreign body. Scout images of the CT brain show a ring-like object adjacent to the skull vertex and an anterior fontanelle. Brain computed tomography (CT) with a bone window revealed no skull defects. The skull was intact, with no signs of new or healed fractures, as seen on the scan (Figure 1).

However, the patient’s family could not provide a definitive explanation for the presence of a metallic object in the patient’s cranium. A possible explanation for the more anterior location of the object is that it was introduced through the anterior fontanelle during infancy. The neurosurgeon declined the surgical removal of the object due to its position and orientation, as well as the patient’s guardian’s refusal to consent to surgery. Given the potential risks and complexity associated with surgical removal, a conservative management approach was employed, which involved the administration of lifelong anti-seizure medications and precautions against magnetic resonance imaging.

## Discussion

Infections affecting the central nervous system and traumatic brain injuries are leading causes of symptomatic epilepsy. Seizures are more likely to occur following cerebral infection; however, the magnitude and duration of these convulsions remain unclear [5]. The present case is unique in nature because the patient had no history of craniotomies, road traffic accidents, or penetrating head injuries. The presence of a metallic ring near the vertex suggests that it was inserted into the cranial cavity at a young age before anterior fontanel closure. The presence of metallic foreign bodies in the skulls of newborns has been associated with cases of infanticide or murder. However, in other cases, the entry points of the needles were found to be the vertex, nostril, or orbit, indicating accidental introduction [6]. However, in the present case, radiological findings demonstrated that an intracranial foreign object resembled a ring, which is unusual.

Despite being a rare presentation in the medical literature, it is proposed that a stepmother or father, babysitter, or caretakers with a poor psychiatric history [1] are risk factors. Being born into an extramarital affair in a culture that allows the homicide of such children and being the youngest or the female child are factors that make the subject vulnerable [6]. Comprehensive radiographic skeletal surveys should be included in the evaluation when investigating suspected child abuse cases [7] or penetrating intracranial injuries [8].

In the present case, the patient and his family were unable to provide a clear explanation of the presence of a metallic object within an intact skull. No signs of cranial injury or trauma were observed (Figure 1). The mother was unable to recall any incidents in which the child was beaten or injured during infancy or by toddlers. The patient had no history of bleeding wounds in the head or penetration of an object through the skull. He had a history of seizures and has received antiepileptic treatment since infancy. However, it appears that the primary healthcare provider has never conducted a radiographic assessment, such as a CT scan, which could have detected the presence of a foreign body. The likelihood that the origin of the seizures was not discovered during the examination suggests a missed opportunity for discovery. We are presenting this case to emphasize the importance of early diagnosis of a fatal condition like epilepsy and the use of radiographic techniques like a CT scan to rule out the cause of the condition.

Many cases have been reported in the literature where a foreign object, such as a needle, was retained for long periods without presenting any symptoms [3]. Epilepsy and status epilepticus are usually the symptoms when intracranial objects present late in life, and sudden-onset seizures are typical symptoms of both recent and reclusive penetrating injuries [9]. Late-onset seizures can be attributed to slow gliosis, progressive granulomatous changes, prolonged abscess formation, and metal toxicity in cases of retained foreign bodies [5]. Numerous studies have shown that surgery may not be required when seizures can be managed by medication, as long as there is no risk of infection and the diagnosis is purely incidental [9,10]. In this particular case, the patient was symptomatic and considered a potential candidate for surgical removal of the object through craniotomy. The neurosurgery team conducted a thorough examination and a comprehensive investigation to evaluate the need for surgical intervention. However, the team discouraged surgical removal by considering the object's location. Extensive surgical intervention is required to remove these objects. The patient was a minor who required consent for surgical removal from guardians who were unwilling to proceed with this option. Moreover, the author of this case report observed that the patient experienced recurrent seizures because he frequently missed his medication and had poor medication compliance. Moreover, his mother shared that he does not experience seizures when he takes his medicine regularly. Therefore, we decided to continue with the present regime. The patient and his parents were informed of the possible adverse effects of noncompliance. The severity of the condition (worsening of the symptoms, need for frequent hospitalization, and even death) and the long-term effects of taking antiepileptic drugs (nausea, vomiting, diarrhea, weakness, headaches, and depression) were explained to the patients. Patients were encouraged to adhere to the follow-up schedule. The development of infections attributable to the presence of a foreign object should be carefully considered and ruled out through a prolonged follow-up.

Limitations: The patient’s family had limited recollection of the child’s history of seizures or any cranial injury, leading to poor recall and a lack of detailed information. Additionally, there is no document record that could provide information regarding the duration of valproic acid treatment, posing a challenge in selecting an appropriate treatment regimen and adjusting the dosage of medicines. The healthcare professionals’ team was unable to establish a definitive cause for the presence of the foreign body owing to an intact skull and an incomplete and unclear history provided by the family regarding previous traumatic events.

## Conclusions

This case emphasizes the significance of thorough history-taking and evaluation in patients with a history of seizures, as well as the potential utility of advanced imaging techniques, such as CT scans, in identifying incidental foreign bodies in the cranial cavity. Although there have been previous reports of sewing needles being introduced through the anterior fontanelle, to our knowledge, this is the first case of a ring-like metallic object within the cranium. The decision to pursue surgical removal should be based on careful consideration of potential risks and benefits.
